# NMR Dynamic View of the Stabilization of the WW4 Domain by Neutral NaCl and Kosmotropic Na_2_SO_4_ and NaH_2_PO_4_

**DOI:** 10.3390/ijms25169091

**Published:** 2024-08-22

**Authors:** Liang-Zhong Lim, Jianxing Song

**Affiliations:** Department of Biological Sciences, Faculty of Science, National University of Singapore, 10 Kent Ridge Crescent, Singapore 119260, Singapore

**Keywords:** Hofmeister series, kosmotropic ion, protein conformation, thermodynamic stability, protein dynamics, NMR spectroscopy

## Abstract

The Hofmeister series categorizes ions based on their effects on protein stability, yet the microscopic mechanism remains a mystery. In this series, NaCl is neutral, Na_2_SO_4_ and Na_2_HPO_4_ are kosmotropic, while GdmCl and NaSCN are chaotropic. This study employs CD and NMR to investigate the effects of NaCl, Na_2_SO_4_, and Na_2_HPO_4_ on the conformation, stability, binding, and backbone dynamics (ps-ns and µs-ms time scales) of the WW4 domain with a high stability and accessible side chains at concentrations ≤ 200 mM. The results indicated that none of the three salts altered the conformation of WW4 or showed significant binding to the four aliphatic hydrophobic side chains. NaCl had no effect on its thermal stability, while Na_2_SO_4_ and Na_2_HPO_4_ enhanced the stability by ~5 °C. Interestingly, NaCl only weakly interacted with the Arg27 amide proton, whereas Na_2_SO_4_ bound to Arg27 and Phe31 amide protons with Kd of 32.7 and 41.6 mM, respectively. Na_2_HPO_4_, however, bound in a non-saturable manner to Trp9, His24, and Asn36 amide protons. While the three salts had negligible effects on ps-ns backbone dynamics, NaCl and Na_2_SO_4_ displayed no effect while Na_2_HPO_4_ significantly increased the µs-ms backbone dynamics. These findings, combined with our recent results with GdmCl and NaSCN, suggest a microscopic mechanism for the Hofmeister series. Additionally, the data revealed a lack of simple correlation between thermodynamic stability and backbone dynamics, most likely due to enthalpy–entropy compensation. Our study rationalizes the selection of chloride and phosphate as the primary anions in extracellular and intracellular spaces, as well as polyphosphate as a primitive chaperone in certain single-cell organisms.

## 1. Introduction

Around half of the human proteome must adopt specific three-dimensional structures to function in cells [1]. Proteins are characterized by different thermodynamic stabilities, referring to the free energy difference between their folded and unfolded states [2,3,4], as well as inherent dynamics involving structural motions and fluctuations across various time scales [5,6,7]. It is well established that protein functions, interactions with other molecules, and aggregation accountable for diverse diseases are governed not only by structures, but also by stability and dynamics [2,3,4,5,6,7]. However, understanding the microscopic interactions and mechanisms, as well as the relationships among protein structures, thermodynamic stability, and dynamics, remains a fundamental challenge in protein science.

**Figure 1:**
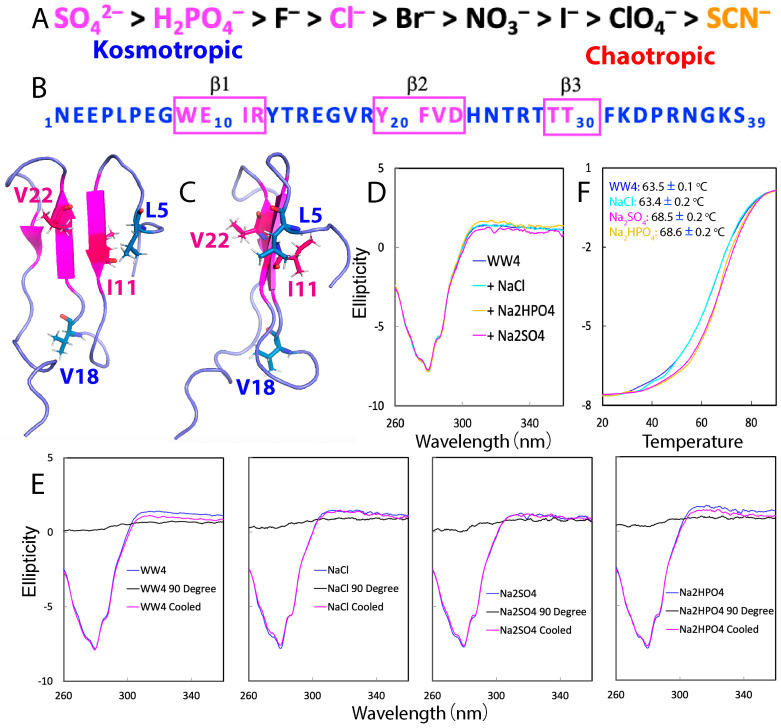
Effects of the three salts on the tertiary structure and thermal stability of WW4. (**A**) The Hofmeister series of common anions. (**B**) Sequence of the WW4 domain with the amino acids in the β-strands, boxed and colored in purple. (**C**) NMR structure of WW4 (PDB code of 2OP7), with four hydrophobic residues displayed in sticks. (**D**) Near-UV CD spectra of WW4 in the absence and in the presence of NaCl, Na_2_SO_4_, and Na_2_HPO_4_, respectively, at 200 mM. (**E**) Near-UV CD spectra of WW4 recorded under four different conditions at 25 °C, at 90 °C, and after cooling back down to 25 °C following thermal unfolding. (**F**) Thermal unfolding curves of ellipticity at 280 nm under four conditions.

Recently, through deep learning of existing structures, artificial intelligence (AI) systems, such as AlphaFold 2 and 3 using neural network architectures, have gained the ability to accurately predict the structures of proteins and their complexes without requiring knowledge of their microscopic interactions or mechanisms [8,9]. On the other hand, despite exhaustive studies for more than 100 years [1,2,3,4,5,6,7], the mechanisms and relationship between folding, thermodynamic stability, and dynamics of proteins remain not fully understood. One powerful method to explore these key issues is to experimentally measure protein conformation, thermodynamic stability, and dynamics in the presence of salts, including denaturants and stabilizers. The Hofmeister series, established over 130 years ago by the pioneering work of Franz Hofmeister, classifies ions based on their effects on protein stability in aqueous solutions [4,10,11,12,13,14,15,16,17]. Ions that enhance protein stability are termed kosmotropes, whereas those that reduce stability are known as chaotropes. As shown in Figure 1A, within the series, Na_2_SO_4_ and Na_2_HPO_4_ are strong kosmotropes, while GdmCl and NaSCN are strong chaotropes. NaCl is located in the middle and considered neutral. The Hofmeister effects are universally observed across a wide range of disciplines, including medicine, biology, chemistry, and industrial science. Despite their pervasive presence, the precise microscopic mechanisms underlying the Hofmeister series remain largely unclear. It is generally believed that these effects arise from complex and specific interactions between ions and proteins, as well as between ions and the water molecules directly surrounding proteins, affecting protein hydration. However, the inherent complexity of systems involving ions, counterions, solvents, and co-solutes, each playing different roles, makes it a major challenge to elucidate the detailed microscopic mechanisms [10,11,12,13,14,15,16,17].

NMR studies on protein conformation, stability, binding, and dynamics can provide detailed insights into how Hofmeister series ions influence protein stability. However, two key challenges exist: (1) Hydrophobic side chains in most proteins remain buried and inaccessible to ions at low concentrations before global unfolding occurs. (2) However, upon global unfolding, the resulting changes in protein NMR resonances are caused by both conformational changes and ion–protein interactions, making it difficult to distinguish between the two effects. In this context, we recently selected a folded 39-residue WW4 domain without any Cys residue (Figure 1B) and evaluated the effects of the chaotropic salts GdmCl and NaSCN on its conformation, thermal stability, binding, and backbone dynamics at low salt concentrations (≤200 mM) by CD and NMR spectroscopy [18]. The WW4 domain from the ubiquitin ligase WWP1 adopts the classic WW fold (2OP7), as determined by NMR, and shows no detectable self-association even at concentrations up to 2 mM, as characterized by 1D ^1^H NMR spectroscopy [18,19]. Most importantly, unlike large-folded proteins with hydrophobic side chains buried in the folded state, WW4 consists of a flat three-stranded β-sheet, making its hydrophilic and hydrophobic residues largely accessible even in the native state (Figure 1C). This accessibility allows WW4 residues to interact with salt ions even at low concentrations, before global unfolding occurs. Consequently, any binding events should be detectable by 1D and HSQC NMR spectroscopy, which can resolve residue-specific interactions and perturbations across a wide range of affinities, including the dissociation constant (Kd) in the millimolar range [20,21,22,23,24,25,26]. The results indicate: (1) Up to 200 mM, both denaturants did not affect the tertiary structure of WW4, but GdmCl caused more destabilization than NaSCN. (2) GdmCl only weakly bound to amide protons, whereas NaSCN extensively bound to both hydrophobic side chains and amide protons of Arg27, Thr28, Thr29, and Thr30, with Kd ranging from 62.4 to 108.6 mM, respectively. (3) Neither denaturant significantly impacted ps-ns backbone dynamics, but they distinctly affected μs-ms backbone dynamics.

In this study, to ensure that the obtained results could be directly compared to the previous findings with NaSCN and GdmCl [18], we employed the same CD and NMR experimental settings to evaluate the effects of three additional salts on WW4 under identical conditions: NaCl, considered neutral in the Hofmeister series, and the strong kosmotropic Na_2_SO_4_ and Na_2_HPO_4_ (Figure 1A). Our findings revealed: (1) Up to 200 mM, all three salts showed no detectable impact on the tertiary structure of WW4 or on the reversibility of its thermal unfolding. (2) At 200 mM, NaCl had no effect on the thermal stability of WW4, whereas Na_2_SO_4_ and Na_2_HPO_4_ increased its melting temperature (Tm) by ~5 °C. (3) None of the salts showed significant binding to hydrophobic side chains. NaCl exhibited minor, non-saturable binding to Arg27, while Na_2_SO_4_ bound to Arg27 and Phe31 with Kd values of 32.7 ± 5.1 mM and 41.6 ± 4.3 mM, respectively. Na_2_HPO_4_ bound to Trp9, His24, and Asn36 in a non-saturable manner. (4) The three salts had no significant effects on ps-ns backbone dynamics. While NaCl and Na_2_SO_4_ did not significantly affect μs-ms backbone dynamics, Na_2_HPO_4_ markedly increased μs-ms backbone dynamics even at 20 mM. These results, together with our previous findings on GdmCl and NaSCN [18], shed light on potential mechanisms underlying the Hofmeister series and underscore the lack of a straightforward correlation between thermodynamic stability and backbone dynamics at both ps-ns and μs-ms time scales.

## 2. Results

### 2.1. CD Characterization of the Effects of NaCl, Na_2_SO_4_, and Na_2_HPO_4_ on WW4

As the presence of three salts at high concentrations would generate very high non-specific noise over the far-UV CD region, here, we assessed the effects of NaCl, Na_2_SO_4_, and Na_2_HPO_4_ on the conformation and thermal stability of WW4 by using the near-UV CD region over 260–360 nm, which reports the protein tertiary structure, exactly as we have recently monitored the effects of GdmCl or NaSCN on WW4 [18]. As shown in Figure 1D, the negative signal in the near-UV spectra at 280 nm suggested that the WW4 domain, although quite small, possessed a tightly packed tertiary structure. Additionally, the near-UV CD spectra remained nearly identical in the absence and presence of NaCl, Na_2_SO_4_, and Na_2_HPO_4_ at 200 mM, indicating that the tertiary structure of WW4 was not significantly affected by any of the three salts. Next, we assessed the thermodynamic stability of WW4 under four different conditions by conducting thermal unfolding experiments on the Cys-free WW4 variant, as we recently conducted on WW4 with GdmCl and NaSCN [18]. Figure 1E shows the CD spectra of WW4 at 25 °C and 90 °C, with and without 200 mM of NaCl, Na_2_SO_4_, and Na_2_HPO_4_, as well as the spectra of the samples after being cooled back to 25 °C post-thermal unfolding. The results indicated the following: (1) At 90 °C, the tertiary structure of WW4 was completely disrupted under all four conditions. (2) However, upon cooling the three unfolded samples back to 25 °C, WW4 refolded, and the native tertiary structure was fully reinstated. (3) The unfolding of WW4, both in the absence and in the presence of NaCl, Na_2_SO_4_, and Na_2_HPO_4_ at 200 mM, was found to be all reversible. This behavior sharply contrasts with previous observations on the 87-residue RNA-recognition motif (RRM) domain of the ALS-causing FUS protein, which, despite lacking Cys residues, exhibited dynamic self-association during thermal unfolding, leading to an irreversible thermal denaturation process [25].

Additionally, Figure 1F presents the unfolding profiles, represented by changes in ellipticity at 280 nm as the temperature increased from 20 to 90 °C for WW4 in the absence and presence of 200 mM of NaCl, Na_2_SO_4_, and Na_2_HPO_4_. Despite its small size of only 39 residues and lacking any disulfide bridges and cofactors, WW4 demonstrated a notably high melting temperature (Tm) of 63.5 ± 0.1 °C. This Tm surpasses that of many larger folded proteins, such as the 87-residue RRM domain of FUS with a Tm of ~52 °C [25], and the 140-residue human profilin 1 (hPFN1) with a Tm of ~56 °C [26]. Interestingly, the unfolding curve of WW4 in the presence of 200 mM of NaCl was almost identical to that without any salts, indicating that NaCl did not affect the thermal stability of WW4. By contrast, Na_2_SO_4_ and Na_2_HPO_4_ at 200 mM did enhance thermal stability. Specifically, the melting temperature of WW4 increased from ~63.5 ± 0.1 °C to ~68.5 ± 0.2 °C and ~68.6 ± 0.2 °C with 200 mM of Na_2_SO_4_ and Na_2_HPO_4_, respectively (Figure 1E). These results clearly showed that Na_2_SO_4_ and Na_2_HPO_4_ could raise the melting temperature by ~5 °C, whereas NaCl had no effect, aligning perfectly with the classic Hofmeister ranking (Figure 1A). Thermal unfolding measurements of WW4 in the presence of 20 mM of Na_2_SO_4_ and Na_2_HPO_4_ showed unfolding curves very similar to those without any salts, indicating no detectable impact on WW4’s stability at this lower concentration.

### 2.2. NMR Characterization of the Binding of NaCl, Na_2_SO_4_, and Na_2_HPO_4_ with WW4

**Figure 2:**
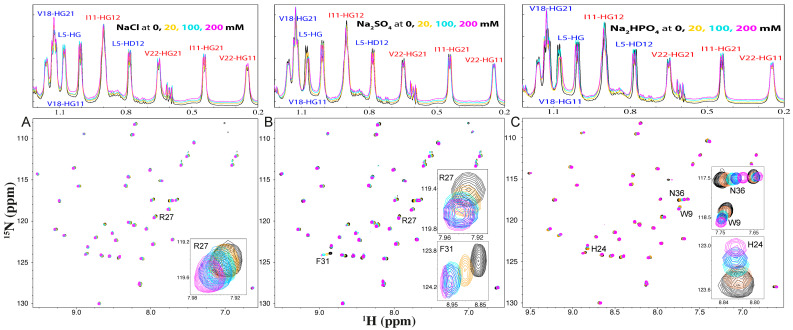
Characterization of the binding of the three salts to WW4 by NMR. Perturbations to 1D NMR resonance peaks of the side chains of Leu5, Ile11, Val18, and Val22, and HSQC peaks of WW4 in the absence (black) and in the presence of NaCl (**A**), Na_2_SO_4_ (**B**), and Na_2_HPO_4_ (**C**) at 20 mM (brown), 100 mM (cyan), and 200 mM (purple). Inlets: the peak tracings of the residues with significant shifts in the absence (black) and in the presence of different salts at 20 mM (brown), 100 mM (cyan), 150 mM (blue), and 200 mM (purple).

We also evaluated the interaction of NaCl, Na_2_SO_4_, and Na_2_HPO_4_ with WW4 using 1D and HSQC NMR spectroscopy. Notably, the addition of these salts, even at concentrations up to 200 mM, did not cause significant changes in the chemical shifts of the methyl or methylene resonances of the four aliphatic hydrophobic acids, including Leu5, Ile11, Val18, and Val22, in the 1D NMR spectra (Figure 2). This suggests that there was no detectable binding of the three salts to these hydrophobic side chains.

**Figure 3:**
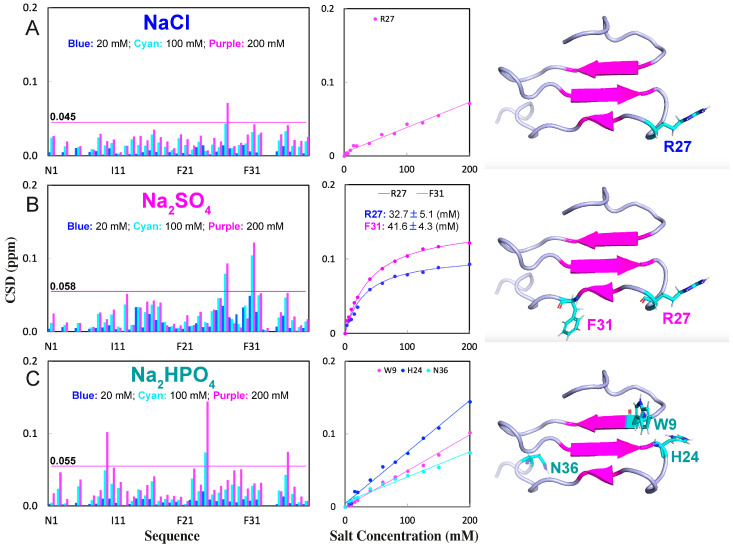
Quantification of the binding of the three salts to WW4 by NMR. Chemical shift difference (CSD) in HSQC peaks of WW4 at 20 mM (blue), 100 mM (cyan), and 200 mM (purple), shift tracings for residues with the significant shift (CSD > average + STD), as well as these residues mapped back to the WW4 structure upon addition of NaCl (**A**), Na_2_SO_4_ (**B**), and Na_2_HPO_4_ (**C**). The purple lines have the indicated values, which are the sums of the average and STD of CSD in the presence of the three salts at 200 mM.

Additional HSQC titrations revealed that NaCl only weakly interacted with some exposed backbone and side-chain amide protons (Figure 2A and Figure 3A). Although at 200 mM, NaCl induced shifts in a set of HSQC peaks, including that of Asn36, only the peak of Arg27 had a significant shift, whose chemical shift difference (CSD) values exceeded the average plus standard deviation (Figure 2A and Figure 3A). On the other hand, the addition of Na_2_SO_4_ resulted in relatively large shifts in many amide protons (Figure 2B and Figure 3B). Notably, the HSQC peaks of Arg27 and Phe31 showed significant shifts. Interestingly, the addition of Na_2_HPO_4_ also triggered relatively large shifts of many amide protons (Figure 2C and Figure 3C). However, three different residues, Trp9, His24, and Asn36, had HSQC peaks with significant shifts (Figure 3C).

For a folded protein, the salt-induced changes of its NMR signals may result directly from the binding interaction, or/and indirectly from the alterations of conformations and dynamics [20,21,22,23,24,25,26]. Here, the three salts with concentrations up to 200 mM showed no detectable effects on the tertiary structure of WW4, as reported by near-UV spectra (Figure 1D) and reflected by no significant change in HSQC spectral dispersions of WW4 (Figure 2). As such, the shifts observed in the HSQC peaks of WW4 that occurred upon adding three salts were expected to primarily result from direct binding interactions of the salts with the WW4 residues.

Interestingly, the shift tracings of Arg27 induced by NaCl, as well as Trp9, His24, and Asn36 by Na_2_HPO_4_, were all non-saturable, while the shift tracings of Arg27 and Phe31 induced by Na_2_SO_4_ were saturable. This is likely because the binding affinity of Na_2_SO_4_ to protein amide protons was much higher than those of NaCl and Na_2_HPO_4_, as we previously found in an intrinsically disordered domain of ephrin-B2 [22]. By assuming that the shifts in HSQC peaks were mostly from the direct binding with salt molecules, the dissociation constants (Kd) with the addition of Na_2_SO_4_ were fitted out from the shift tracing data to be 32.7 ± 5.1 mM and 41.6 ± 4.3 mM, respectively, for Arg27 and Phe31 (Figure 3B).

By comparing the current results with our previous results with GdmCl and NaSCN under the same experimental condition [18], several findings were revealed: (1) NaCl and GdmCl had very similar binding patterns with Arg27, having a significant shift in the HSQC peaks, implying that the shifts may mainly result from the interaction of Cl^−^ anion with WW4 residues. (2) NaCl had no detectable effect on the thermal stability, while GdmCl reduced the Tm of WW4 by ~9 °C, implying that the denaturing capacity of GdmCl was mostly from the Gdm^+^ cation. (3) NaCl, Na_2_SO_4_, and Na_2_HPO_4_ all failed to bind to the four hydrophobic side chains of WW4, while NaSCN could bind to the hydrophobic side chains of WW4 [18]. (4) Interestingly, Na_2_SO_4_ and Na_2_HPO_4_ bound to amide protons of residues, which is highly different from the results of NaSCN. NaSCN bound to Arg27, Thr28, Thr29, and Thr30, which appeared to be important for stabilizing proteins, as previously found in the 37-residue small protein, in which Thr residues also participated in forming the hydrophobic core [27,28,29]. As NaCl, Na_2_SO_4_, Na_2_HPO_4_, and NaSCN all share the same Na^+^ cation, their differences in interacting with WW4 are thus expected to mostly result from anions.

### 2.3. Effects of NaCl, Na_2_SO_4_, and Na_2_HPO_4_ on ps-ns Backbone Dynamics of WW4

We then decided to assess the dynamic effects of NaCl, Na_2_SO_4_, and Na_2_HPO_4_ on WW4 by acquiring a large set of ^15^N backbone relaxation data, including longitudinal relaxation time T1, transverse relaxation time T2, and {^1^H}-^15^N steady-state NOE (hNOE). Initially, we acquired data for WW4 in the absence and in the presence of 200 mM of NaCl, 200 mM of Na_2_SO_4_, and 200 mM of Na_2_HPO_4_. However, after analysis of the data, we found that there existed very high Rex, indicative of conformational exchanges on the µs-ms time scale for WW4 in the presence of 200 mM of Na_2_HPO_4_. We thus further collected the data of WW4 in the presence of 20 mM of Na_2_HPO_4_.

**Figure 4:**
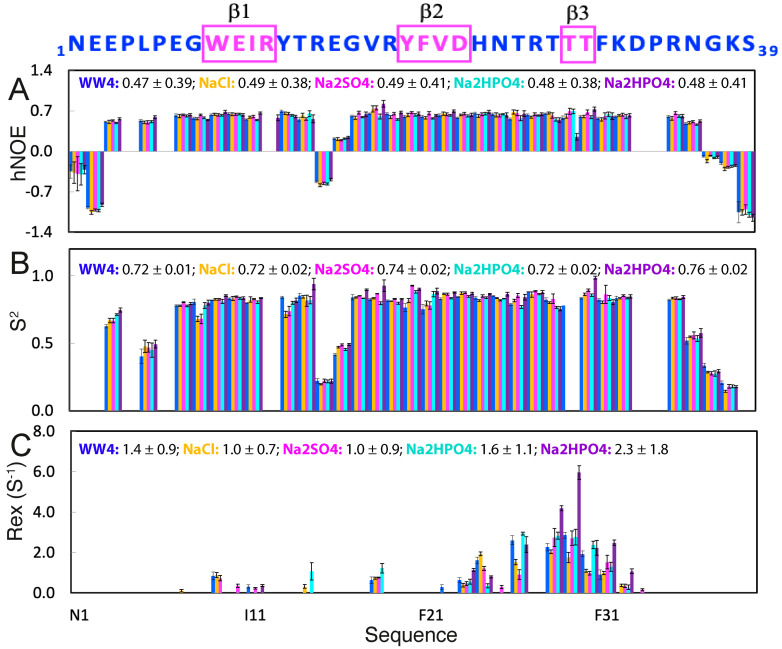
Effects of the three salts on the backbone dynamics of WW4. (**A**) hNOE values of WW4 in the absence (blue) and in the presence of NaCl at 200 mM (brown), Na_2_SO_4_ at 200 mM (purple), and Na_2_HPO_4_ at 20 mM (cyan), as well as Na_2_HPO_4_ at 200 mM (deep purple). (**B**) Squared generalized order parameters (S^2^) of WW4 in the absence (blue) and in the presence of NaCl at 200 mM (brown), Na_2_SO_4_ at 200 mM (purple), and Na_2_HPO_4_ at 20 mM (cyan), as well as Na_2_HPO_4_ at 200 mM (deep purple). (**C**) Residue-specific Rex of WW4 in the absence (blue) and in the presence of NaCl at 200 mM (brown), Na_2_SO_4_ at 200 mM (purple), and Na_2_HPO_4_ at 20 mM (cyan), as well as Na_2_HPO_4_ at 200 mM (deep purple).

Figure 4A presents hNOE data of WW4 under five conditions, which offers a reliable measure of backbone dynamics on the ps-ns time scale [25,26,30,31,32,33,34,35,36,37,38,39]. The similar hNOE values indicated that WW4 had no significant change in ps-ns backbone dynamics under all five conditions. We then used “Model-Free” formalism to analyze the data [25,26,31,32,33,34,35,36,38,39] with the program Dynamics 3 [33], which includes several extended models in addition to the classic “Model-Free approach”. Figure 4B presents the squared generalized order parameters, S^2^, of WW4 under five conditions, which reflect ps-ns conformational dynamics, ranging from 0 for high internal motion, to 1 for completely restricted motion. Except for the terminal residues Asn1-Leu5 and Asn36-Ser39, and the loop residues Arg15-Glu16, the other non-Proline residues all had S^2^ > 0.7 (Figure 4B), indicating that WW4 was well folded. On the other hand, addition of the three salts only resulted in some slight alterations of the residue-specific S^2^ values (Figure 4B), implying slight redistributions of ps-ns backbone dynamics. Nevertheless, average S^2^ values were very similar under all five conditions: 0.72 ± 0.01 (no salt), 0.72 ± 0.02 (200 mM NaCl), 0.74 ± 0.02 (200 mM Na_2_SO_4_), 0.72 ± 0.02 (20 mM Na_2_HPO_4_), and 0.76 ± 0.02 (200 mM Na_2_HPO_4_). The results suggested that the addition of the three salts even up to 200 mM did not significantly alter the overall backbone dynamics of WW4 on the ps-ns time scale.

### 2.4. Effects of NaCl, Na_2_SO_4_, and Na_2_HPO_4_ on µs-ns Backbone Dynamics of WW4

Analysis by Dynamics 3 [33] also indicated that some WW4 residues have additional Rex, which reflects conformational exchanges on the µs-ms time scale. As shown in Figure 4C, without salt, residues with Rex included Trp9, Ile11, Val18, Val22, Asp23, His24, Thr26, Thr28, Thr29, Thr30, and Phe31. In particular, His24, Thr26, Thr28, Thr29, and Thr30 had Rex > 1 Hz. Markedly, the addition of the three salts at different concentrations did not significantly change the overall patterns, but it did alter the Rex values (Figure 4C). For example, the addition of NaCl at 200 mM reduced the average Rex value from 1.4 ± 0.9 (no salt) to 1.0 ± 0.7 (Hz), while the addition of Na_2_SO_4_ at 200 mM reduced the average Rex value to 1.0 ± 0.9 (Hz). Very unexpectedly, the addition of Na_2_HPO_4_ at 20 mM increased the average Rex value to 1.6 ± 1.1, while the addition of Na_2_HPO_4_ at 200 mM dramatically increased the average Rex value to 2.3 ± 1.8.

**Figure 5:**
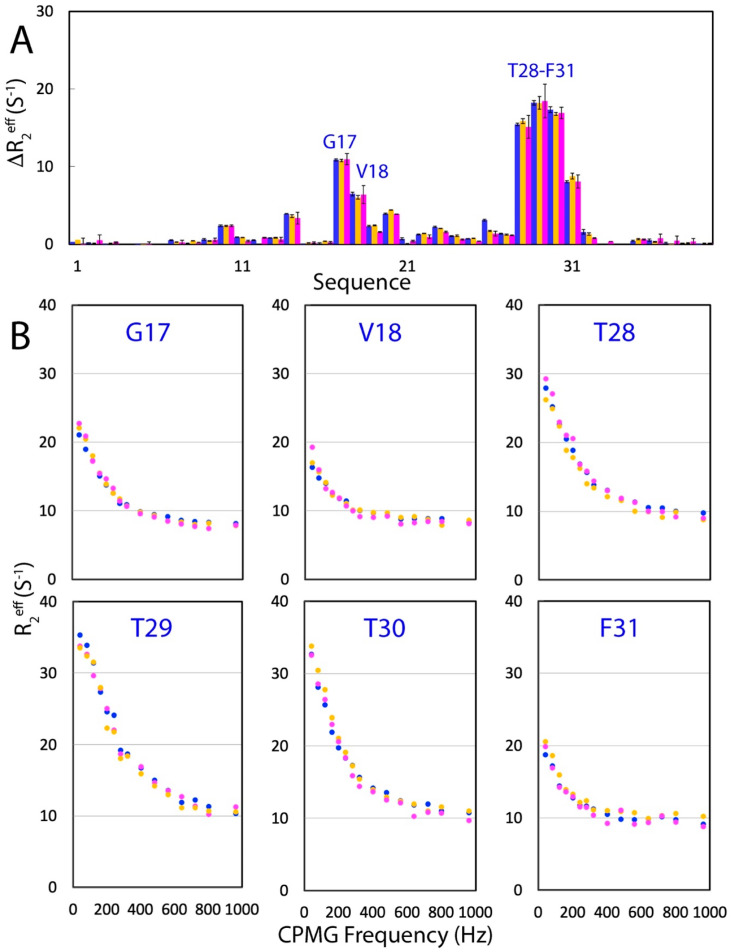
Effects of NaCl and Na_2_SO_4_ on ^15^N backbone CPMG relaxation dispersion. (**A**) Differences in effective transverse relaxation rates (ΔR_2_^eff^) at 80 and 960 MHz for WW4 in the absence (blue) and in the presence of NaCl at 200 mM (brown) and Na_2_SO_4_ at 200 mM (purple) at the 500 MHz field. (**B**) Dispersion curves for 6 WW4 residues in the absence (blue) and in the presence of NaCl at 200 mM (brown) and Na_2_SO_4_ at 200 mM (purple) at the 500 MHz field.

To independently confirm the effects of the three salts on the µs-ms backbone dynamics of WW4, we further performed ^15^N backbone CPMG relaxation dispersion measurements of WW4 under five conditions at 500 MHz magnetic fields [36,40,41,42,43,44,45,46]. Figure 5A presents ΔR_2_^eff^, the differences in effective transverse relaxation rates at 80 and 960 Hz for WW4 in the absence and in the presence of NaCl and Na_2_SO_4_ at 200 mM. Briefly, the patterns of ΔR_2_^eff^ showed no significant differences under the three conditions. The pattern was, in general, consistent with that of Rex derived from “Model-Free” analysis. However, His24 and Thr26 with large Rex values showed no significant CPMG dispersion response. This is commonly observed in a variety of NMR dynamics studies, and many mechanisms might exist to account for the discrepancy between two measurements [36,40,41,42,43]. Furthermore, we analyzed the CPMG dispersion profiles of residues with ΔR_2_^eff^ > 4 Hz, which included Arg17, Val28, Thr28, Thr29, Thr30, and Phe31 (Figure 5B). In general, the six residues had very similar CPMG dispersion profiles under the three conditions, clearly indicating that the presence of NaCl and Na_2_SO_4_ at 200 mM had no significant effects on the µs-ns backbone dynamics of WW4, as measured by CPMG dispersion experiments.

**Figure 6:**
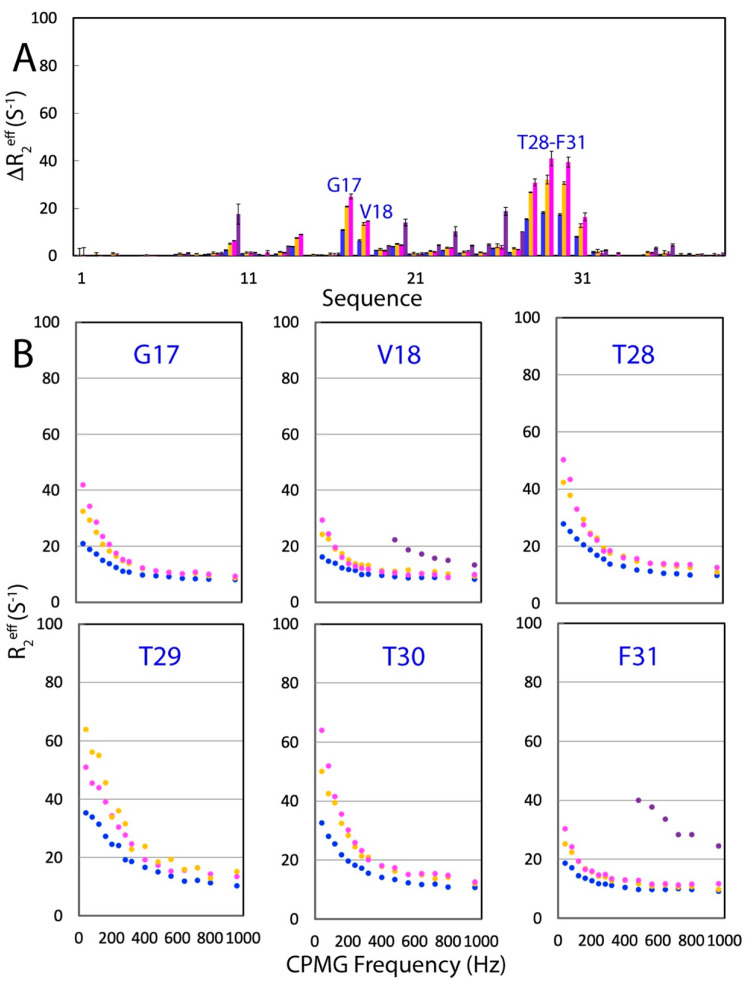
Effects of Na_2_HPO_4_ on ^15^N backbone CPMG relaxation dispersion. (**A**) Differences in effective transverse relaxation rates (ΔR_2_^eff^) at 80 and 960 MHz for WW4 in the absence (blue) and in the presence of Na_2_HPO_4_ at 20 mM (brown) and 200 mM (purple) at the 500 MHz field, as well as Na_2_HPO_4_ at 20 mM (deep purple) at the 800 MHz field. (**B**) Dispersion curves for 6 WW4 residues in the absence (blue) and in the presence of Na_2_HPO_4_ at 20 mM (brown) and 200 mM (purple) at the 500 MHz field, as well as Na_2_HPO_4_ at 20 mM (deep purple) at the 800 MHz field.

Most unexpectedly, however, the addition of Na_2_HPO_4_ significantly increased the ΔR_2_^eff^ values of WW4 residues even at the 500 MHz field (Figure 6). The addition of Na_2_HPO_4_ increased the ΔR_2_^eff^ values from 10.87 Hz (without salt) to 20.78 Hz (20 mM) and to 24.96 Hz (200 mM) for Arg17, from 6.48 Hz (without salt) to 13.42 Hz (20 mM) and to 14.61 Hz (200 mM) for Val18, from 15.44 Hz (without salt) to 26.75 Hz (20 mM) and to 30.84 Hz (200 mM) for Thr28, from 18.24 Hz (without salt) to 32.08 Hz (20 mM) and to 40.98 Hz (200 mM) for Thr29, from 17.34 Hz (without salt) to 30.54 Hz (20 mM) and to 39.41 Hz (200 mM) for Thr30, and from 8.04 Hz (without salt) to 12.72 Hz (20 mM) and to 16.39 Hz (200 mM) for Phe31 (Figure 6A). We also acquired CPMG relaxation dispersion data of WW4 in the presence of Na_2_HPO_4_ at 20 mM at the 800 MHz field. However, the peak intensities of Arg17, Thr28, Thr29, and Thr30 were too weak to calculate reliable R_2_^eff^ values at all CPMG frequencies. For Val18 and Phe31, their peak intensities with CPMG frequencies ≤ 400 Hz were also too weak to calculate reliable R_2_^eff^ values (Figure 6B). As we could not obtain CPMG data at the 800 MHz field even for WW4 in the presence of Na_2_HPO_4_ at 20 mM, we did not proceed to fit the CPMG dispersion data to obtain quantitative exchange parameters.

Although NMR relaxation and CPMG relaxation dispersion measurements can be influenced by self-association [45,46], this did not seem to be the case for the present results with WW4 in the presence of different salts, as (1) it showed no concentration-dependent changes in NMR signals at protein concentrations up to 2 mM [18,19]. (2) We conducted quick CPMG relaxation dispersion measurements on WW4 in the free state even at 2.0 mM but found no significant alterations in ΔR_2_^eff^ values. (3) The relatively large changes in R2 values in the presence of NaCl, Na_2_SO_4_, Na_2_HPO_4_, GdmCl, and NaSCN occurred only over the WW4 residues with significant μs-ms dynamics, while the WW4 residues in the well-folded regions but without significant μs-ms dynamics showed no significant increase in R2 values. (4) In particular, the five salts consisting of denaturants, a neutral one, and stabilizers, despite all being charged ions, induced differential and even opposite changes in both Rex and ΔR_2_^eff^ values, rendering them incompatible with the possibility that the five salts might induce dynamic self-association of WW4 via electrostatic/salt effects.

## 3. Discussion

The remarkable capacity of AI systems in accurately predicting protein structures [8,9] highlights a paradigm shift to focus on understanding protein thermodynamic stability and dynamics. This understanding is crucial not only for the physiological roles of proteins but also for the pathological factors governing protein aggregation associated with various human diseases, including neurodegenerative diseases, cancers, and heart failure [47,48,49,50,51,52,53]. In this context, two pivotal questions emerge: (1) What is the relationship between protein thermodynamic stability and dynamics? (2) How do other molecules influence protein thermodynamic stability and dynamics?

One ubiquitous category of molecules universally influencing protein folding, stability, and dynamics are salts. Although salts are recognized for their functions in modern cell physiology and pathology, salts might also play crucial roles in driving the origin of life. For instance, water bodies were hypothesized to be highly unsalted in the early prebiotic world, allowing the solubilization of proteins/peptides prone to aggregation in salted water [50,54], as well as the formation of ATP, the universal energy currency and modulator of protein hemostasis [55,56,57,58,59,60]. The formation of ATP and subsequent increases in salt concentrations might have driven the formation of protocells, simple precursors to modern cells [60]. Despite their relatively simple chemical structures, salt ions appear to influence proteins through both non-specific electrostatic effects and specific interactions, as exemplified by the Hofmeister series, first recognized in 1888 [10]. However, despite extensive studies, the microscopic mechanisms underlying the Hofmeister series remain a fundamental mystery.

**Figure 7:**
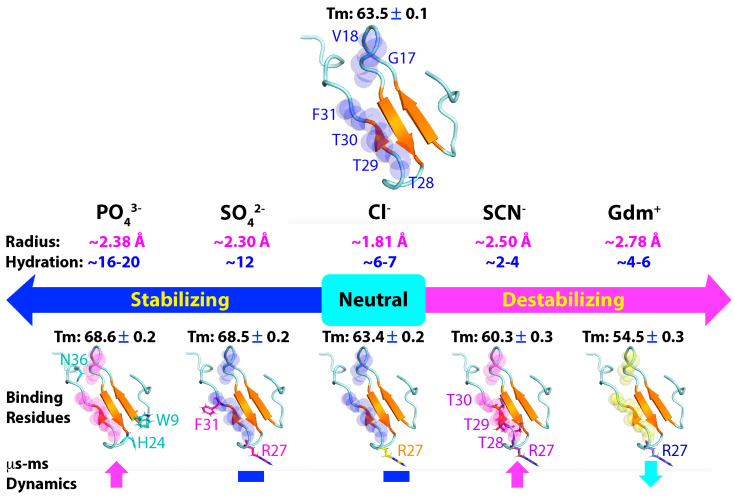
The effects of the four anions and Gdm^+^ cation on the conformation, thermodynamic stability, binding residues, and backbone dynamics of the WW4 domain. The ions are ranked based on the Hofmeister series, with the ionic radius and water molecule numbers upon hydration indicated. The thermodynamic stability is reported by the melting temperature (Tm) values of WW4 in the absence and in the presence of, respectively, the five salts at 200 mM. In the structure of WW4, the spheres are utilized to indicate six residues: Gly17, Val18, Arg27, Thr28, Thr29, and Thr30, with significant μs-ms backbone dynamics (ΔR_2_^eff^ > 4 Hz at the 500 MHz field), while the sticks are used to show the residues with the significant CSD (CSD > average + STD) in the presence of, respectively, the five salts at 200 mM. The changes in the μs-ms backbone dynamics upon adding, respectively, the five salts at 200 mM are also indicated.

By selecting the WW4 domain, which possesses a high thermodynamic stability and accessible side chains in the native state, we systematically assessed the effects of five salts on its conformation, thermal stability, binding, and backbone dynamics across both ps-ns and μs-ms time scales. These salts included two strong chaotropics, GdmCl and NaSCN [18], the neutral NaCl, as well as the strong kosmotropic Na_2_SO_4_ and Na_2_HPO_4_, all at concentrations ≤ 200 mM, where the volume-excluding effect is negligible (Figure 7). The studies revealed that although up to 200 mM, all five salts caused no detectable alteration in the tertiary structure of WW4, they had differential impacts on the thermal stability: while GdmCl and NaSCN destabilized WW4 by ~9.0 °C and ~3.2 °C, respectively [18], Na_2_SO_4_ and Na_2_HPO_4_ stabilized WW4 by ~5 °C, but NaCl showed no effect. These results are completely consistent with the classic ranking of salts in the Hofmeister series (Figure 1A). On the other hand, at the microscopic level, the five salts exhibited an extreme diversity in binding profiles: (1) Since all salts except GdmCl share the same sodium cation, while GdmCl and NaCl showed similar binding patterns, the diversity in binding profiles most likely resulted from differences in anions, with both sodium and guanidinium cations showing minor binding capacity, consistent with the previous finding on an intrinsically disordered protein [22]. (2) Both sodium (Na^+^) and guanidinium (Gdm^+^) cations showed no significant binding to the four aliphatic hydrophobic side chains, while only SCN^−^, out of the four anions, had the ability to bind to hydrophobic side chains. (3) The four anions displayed diverse binding profiles to amide protons. Briefly, the Cl^−^ anion only showed weak binding to the Arg27 amide proton, while SCN^−^, SO_4_^2−^, and HPO_4_^3−^ bound different sets of residues with varying affinities: SCN^−^ bound Arg27, Thr28, Thr29, and Thr30, SO_4_^2−^ bound Arg27 and Phe31, and HPO_4_^2−^ bound Trp9, His24, and Asn36. Interestingly, only SCN^−^ and SO_4_^2−^ displayed saturable binding profiles, with SO_4_^2−^ having higher affinities than NaSCN.

With regard to ^15^N backbone dynamics of WW4, (1) up to 200 mM, all five salts showed no significant effect on the ps-ns time scale. (2) By contrast, the five salts showed extremely distinctive effects on the µs-ms time scale: NaCl and Na_2_SO_4_ had no significant effects, GdmCl reduced the dynamics, while NaSCN and Na_2_HPO_4_ largely enhanced them. Most notably, even at 20 mM, Na_2_HPO_4_ was able to significantly increase the µs-ms dynamics. (3) Since NaCl had no effect on the µs-ms backbone dynamics, the effect of GdmCl to reduce the µs-ms backbone dynamics most likely came from its guanidinium cation. It is worthwhile to point out that the chloride anion was inert in terms of all these assessed effects. This might be a fundamental reason why nature has selected chloride as the most prevalent and concentrated anion in extracellular fluids, as it is expected to impose minimal perturbations on protein structures and functions.

Our findings of the effects of the five salts on WW4 aligned perfectly with the well-established scenario of the Hofmeister series: despite the consistent ranking of salts’ effects on the thermodynamic stability across various proteins, the underlying microscopic mechanisms were extremely diverse [4,10,11,12,13,14,15,16,17,18,61,62,63,64,65,66,67,68,69,70,71]. This raises a fundamental question: what mechanisms drive this unique phenomenon? The thermodynamic stability of proteins is a complex trait influenced by multiple factors. Key contributors include the hydrophobic effect, electrostatic interactions (such as hydrogen bonds and salt bridges), and the protein’s interaction with water, which forms hydration shells. Each protein exhibits a distinct combination of these factors that collectively determine its stability. The stability is governed by a delicate balance between favorable enthalpic contributions from covalent bonds and non-covalent interactions and unfavorable entropic contributions, primarily due to the loss of conformational entropy upon folding.

In the case of salt ions, their ability to interact with proteins is primarily governed by two key factors: charge density and hydration degree [61,62,63]. As illustrated in Figure 7, the Gdm^+^ cation and SCN^−^ anions have large ionic volumes but low charge numbers, resulting in low charge density. They also exhibit weak hydration degrees, with only 4–6 and 2–4 water molecules surrounding Gdm^+^ and SCN^−^, respectively. Consequently, both ions are considered to disrupt the hydration structure of proteins [61,62,63,64,65]. In contrast, the SO_4_^2−^ and PO_4_^3−^ anions have large ionic volumes and high charge numbers, leading to high charge density. They also possess high hydration degrees, with 12 and 16–20 water molecules surrounding SO_4_^2−^ and PO_4_^3−^, respectively. Thus, the two ions are considered to stabilize the hydration structure of proteins [61,62,63]. The Cl^−^ anion, on the other hand, has a medium ionic volume and low charge number, resulting in medium charge density. Interestingly, it also has a medium hydration degree, with 6–7 water molecules.

In this context, the effects of the five salts on WW4 can be rationalized, as follows: Because NaCl is inert and had no effect on the stability, while both the Gdm^+^ cation and Cl^−^ anion only had minor interactions with WW4, the strong destabilizing effect of GdmCl came from the Gdm^+^ cation, which is capable of strongly disrupting the hydration structure of WW4. This disruption appeared to result in a reduction in the μs-ms backbone dynamics. Similarly, NaSCN appeared to destabilize the stability also via the SCN^−^ anion through breaking the hydration structure of WW4. Additionally, however, the SCN^−^ anion could extensively interact with hydrophobic side chains, thereby disrupting the hydrophobic interactions crucial for protein thermodynamic stability. This disruption is expected to trigger μs-ms backbone dynamics, similar to the increased μs-ms dynamics previously observed by NMR upon the pH-induced disruption of the hydrophobic core of a 37-residue small protein [28,29]. On the other hand, both SO_4_^2−^ and PO_4_^3−^ anions have high charge density and hydration degrees. Consequently, Na_2_SO_4_ and Na_2_HPO_4_ appeared to significantly enhance the thermal stability of WW4 by stabilizing its hydration structure. Additionally, the two anions could also extensively interact with WW4 residues without interacting with hydrophobic side chains. Due to their different properties, Na_2_SO_4_ did not significantly affect the μs-ms backbone dynamics of WW4, whereas Na_2_HPO_4_ significantly enhanced these dynamics.

In this framework, three key factors appeared to govern the capacity and properties of salts to affect protein thermodynamic stability and dynamics: (1) The primary factor was the intrinsic ability of salts to disrupt or stabilize the hydration structure of a protein. (2) An additional factor was the capacity of salt ions to bind with protein residues, which may vary depending on the specific salt and protein involved. (3) The alteration of protein dynamics likely resulted from the interplay between the effect of salts on the hydration structure of the protein and the direct interactions of salts with protein residues. However, the contribution of dynamic changes to thermodynamic stability can be either positive or negative and is influenced by the specific salt and protein through entropy–enthalpy compensation [72,73].

The hydration effect is a universal phenomenon affecting not only proteins but all molecules, providing a rationale for the universality of the Hofmeister series. However, our current understanding of protein hydration, as well as the mechanisms for salts to affect protein hydration, is largely limited due to the significant challenges in experimentally and computationally studying its structures and dynamics [74,75,76,77,78,79,80,81]. Therefore, unraveling the mysteries of water and hydration is crucial for understanding the Hofmeister series. On the other hand, the relationship between protein thermodynamic stability and dynamics represents a fundamental yet contentious subject [81,82,83,84,85]. The present findings with the five different salts suggested that multiple states exist near the native state of WW4. These states own highly similar tertiary structures but exhibit markedly different μs-ms backbone dynamics and thermodynamic stabilities. Consequently, there is no straightforward correlation between the thermodynamic stability and the backbone dynamics of WW4 on the ps-ns and μs-ms time scales. Remarkably, the ability of PO_4_^3−^ to dramatically increase μs-ms backbone dynamics may explain why ATP and triphosphate have the unique capacity to induce the folding of ALS-causing PFN1 and SOD1 mutants [26]. Briefly, as revealed here, the phosphate anion was able to significantly increase μs-ms dynamics, thus facilitating the transition from the unfolded state to the native state separated by relatively low energy barriers. However, for ALS-causing PFN1 and SOD1 mutants with the co-existence of both folded and unfolded states, which are separated by relatively large energy barriers and exchanging on the ms time scale, the capacity of phosphate to induce their folding is too weak and, consequently, diphosphate, triphosphate, or ATP is needed to induce their folding [26]. This may rationalize that in the intracellular spaces, in addition to 10 mM of free phosphate, there are also various phosphate-containing molecules, which include ~45 mM of phosphocreatine, 3.5 mM of hexose phosphate, and 3–12 mM of ATP. While further studies are necessary to explore this possibility, these molecules might also contribute to enhancing the stability and inducing folding of a large array of proteins. It is particularly intriguing to note that nature appears to leverage this mechanism, using polyphosphate as a primitive chaperone to facilitate protein folding in certain single-cell organisms [86,87].

## 4. Materials and Methods

### 4.1. Expression and Purification of WW4

The expression vector for WW4 was previously constructed [19]. For bacterial expression of the recombinant protein, the vector was transformed into *E. coli* BL21 cells. The cells were then cultured at 37 °C until the OD600 reached 0.6. Protein expression was induced by adding IPTG to a final concentration of 0.3 mM, followed by incubation for 12 h at 20 °C. Cells were harvested by centrifugation and lysed by sonication in PBS buffer. The recombinant GST-fused WW4 protein was purified using glutathione-Sepharose 4B beads (Pharmacia Biotech, Piscataway, NJ, USA) under native conditions. The WW4 domain was then cleaved from the GST tag by on-gel thrombin digestion at room temperature for 3 h and further purified using HPLC with a reverse-phase C18 column (Vydac), employing a gradient of water-acetonitrile. For isotope labeling of WW4 for ^1^H-^15^N NMR HSQC experiments, the protein was prepared using a similar protocol, except that the cells were grown in M9 medium supplemented with (^15^NH_4_)_2_SO_4_ for ^15^N labeling [19].

### 4.2. Circular Dichroism (CD) Experiments

Non-specific noise was exceptionally high in the far-UV region in the presence of the three salts. Therefore, in this study, we focused on characterizing the conformation and thermal stability of WW4 by monitoring the near-UV region (260–360 nm). Near-UV CD spectra were collected in 1 mM of Tris-HCl at pH 6.4, both in the absence and presence of varying concentrations of the three salts. The measurements were performed on a Jasco J-810 spectropolarimeter equipped with a thermal controller, as previously described [18,19], using a protein concentration of 250 μM at 25 °C and a 1 mm path length cuvette with a 0.1 nm spectral resolution. Data from five independent scans were combined and averaged. Thermal unfolding was assessed on the same samples, with temperatures ranging from 20 to 90 °C [18].

### 4.3. NMR Titration of NaCl, Na_2_SO_4_, and Na_2_HPO_4_ to WW4

A WW4 stock sample was prepared by dissolving the protein powder in 1 mM of Tris-HCl buffer to a final concentration of 250 μM. The pH was adjusted to 6.4 by adding either diluted sodium hydroxide or hydrochloric acid. This stock was then divided into individual samples for NMR titrations. Three salts were also dissolved in 1 mM of Tris-HCl buffer to 1 M, and their pH values were adjusted to 6.4.

All NMR titration experiments were conducted at 25 °C on an 800 MHz Bruker Avance spectrometer equipped with a shielded cryoprobe, as described previously [18]. During titrations, series of one-dimensional ^1^H and two-dimensional ^1^H-^15^N HSQC spectra were acquired on the ^15^N-labeled WW4 domain at a concentration of 250 μM, both in the absence and presence of varying concentrations of three salts (3, 6, 10, 20, 30, 40, 60, 80, 100, 125, 150, and 200 mM), following our previous methodology with GdmCl and NaSCN [18]. The pH of the NMR samples for each titration was measured before and after the addition of 200 mM of salts, with no detectable differences observed. NMR data were processed using NMRPipe [88] and analyzed with NMRView [89].

### 4.4. Calculation of CSD and Data Fitting to Obtain Kd

To calculate the chemical shift difference (CSD), HSQC spectra were superimposed for the ^15^N-labeled WW4 domain collected in the free state and in the presence of three salts at different concentrations. Subsequently, the shifted HSQC peaks could be identified and further assigned to the corresponding WW4 residues based on the sequential assignment we previously obtained [18,19]. As extensively discussed in [21], the appropriate formula for calculating the integrated index of CSD may vary depending on the specific protein–ligand systems involved [18,19,20,21,22,90,91,92]. For instance, we previously calculated CSD values for separate ^1^H and ^15^N chemical shifts to determine the dissociation constant (Kd) [22]. In the present study, we applied the same formula we had used earlier to characterize the binding of small peptides to this WW4 domain [19]:

CSD = ((Δ^1^H)^2^ + (Δ^15^N/4)^2^)^1/2^ (1)

In order to obtain the residue-specific dissociation constant (Kd), we fitted the shift traces of the residues with large shifts in HSQC peaks (CSD > average + STD) by using the one binding site model with the following formula [21]:

CSD_obs_ = CSD_max_{([P] + [L] + Kd) − [([P] + [L] + Kd)^2^ − 4[P][L]]^1/2^}/2[P] (2)

Here, [P] and [L] are molar concentrations of WW4 and GdmCl and NaSCN, respectively.

### 4.5. NMR Characterization of ^15^N Backbone Dynamics on the ps-ns Time Scale

^15^N backbone T1 and T1ρ relaxation times and {^1^H}-^15^N steady-state NOE intensities were collected for WW4 with a concentration of 250 µM at pH 6.4 under five conditions, namely, WW4 without salt, with 200 mM of NaCl and Na_2_SO_4_, as well as with 20 and 200 mM of Na_2_HPO_4_, on a Bruker DRX 500 MHz spectrometer equipped with pulse-field gradient units at 25 °C [18,93,94]. Relaxation time T1 was determined by collecting HSQC spectra with delays of 10, 80, 200, 320, 360, 420, and 500 ms using a recycle delay of 1 s, with a repeat at 200 ms. Relaxation time T1ρ was measured by collecting spectra with delays of 1, 30, 60, 90, 110, 130, 150, and 180 ms using a spin-lock power of 1.6 kHz and a 2.5 s recycle delay with a repeat at 90 ms. {^1^H}-^15^N steady-state NOEs were obtained by recording spectra with and without ^1^H pre-saturation, a duration of 3 s, and a relaxation delay of 6 s.

NMR relaxation data were analyzed by “Model-Free” formalism with protein dynamics software suites [38,39]. Briefly, relaxation of protonated hetero-nuclei is dominated by the dipolar interaction with the directly attached ^1^H spin and by the chemical shift anisotropy mechanism. Relaxation parameters are given by:

(3) R1=d24JωH−ωX+3JωX+6JωH+ωX+c2JωX

(4) R2=d284J0+JωH−ωX+3JωX+6JωH+6JωH+ωX+c264J0+3JωX+Rex

(5) $$NOE=1+\left(d^{2}/4R_{1}\right)\left(\gamma_{X}/\gamma_{H}\right)\left[6J\left(\omega_{H}+\omega_{X}\right)-J\left(\omega_{H}-\omega_{X}\right)\right]$$

In which $d=\mu_{0}h\gamma_{X}\gamma_{H}\langle r_{XH}^{-3}\rangle /8\pi^{2}$, $c=\omega_{X}\Delta \sigma /\sqrt{3}$, $\mu_{0}$ is the permeability of free space, *h* is Planck’s constant, $\gamma_{X}\;\gamma_{H}$ are the gyromagnetic ratios of ^1^H and the X spin (X = ^13^C or ^15^N), respectively, $\gamma_{XH}$ is the X-H bond length, $\omega_{H}$ and $\omega_{X}$ are the Larmor frequencies of ^1^H and X spins, respectively, and Δσ is the chemical shift anisotropy of the X spin.

The Model-Free formalism, as previously established and further extended [34,35,38,39], determines the amplitudes and time scales of the intramolecular motions by modeling the spectral density function, *J*(*ω*), as:

(6) $$\begin{array}{cc} J\left(\omega \right) & =\frac{2}{5}\left[\frac{S^{2}\tau_{m}}{1+{\left(\omega \tau_{m}\right)}^{2}}+\frac{\left({S_{f}}^{2}-S^{2}\right)\tau }{1+{\left(\omega \tau \right)}^{2}}\right]\\ & =\frac{2}{5}{S_{f}}^{2}\left[\frac{{S_{s}}^{2}\tau_{m}}{1+{\left(\omega \tau_{m}\right)}^{2}}+\frac{\left(1-{S_{s}}^{2}\right)\tau }{1+{\left(\omega \tau \right)}^{2}}\right] \end{array}$$

In which, $\tau =\tau_{s}\tau_{m}/\left(\tau_{s}+\tau_{m}\right)$, $\tau_{m}$ is the isotropic rotational correlation time of the molecule, $\tau_{s}$ is the effective correlation time for internal motions, $S^{2}={S_{f}}^{2}{S_{s}}^{2}$ is the square of the generalized order parameter characterizing the amplitude of the internal motions, and ${S_{f}}^{2}$ and ${S_{s}}^{2}$ are the squares of the order parameters for the internal motions on the fast and slow time scales, respectively.

In order to allow for diverse protein dynamics, several forms of the spectral density function, based on various models of the local motion, were utilized, which included the original Lipari–Szabo approach, assuming fast local motion characterized by the parameters *S*^2^ and *τ_loc_*, extended model-free treatment, including both fast (${S_{fast}}^{2},\;\tau_{fast}$) and slow (${S_{slow}}^{2},\;\tau_{slow}$) reorientations for the NH bond ($\tau_{fast}\ll \tau_{slow}<\tau_{c}$), and could also allow for slow, micro- to milli-second dynamics resulting in a conformational exchange contribution, *R_ex_*.

In the present study, the WW4 NMR structure (2OP7) with the lowest energy was used for “Model-Free” analysis. For HSQC spectra of WW4 under five conditions, all peaks are well-separated and thus data are of high quality, except for the overlap of the Arg12 and Asp33 peaks [19]. Here, the overall rotational diffusion tensors and τ_c_ of WW4 under five conditions were determined by ROTDIF [36], while “Model-Free” analysis of relaxation data was performed using the software Dynamics, which includes the classic and extended “Model-Free” models [34,35]. τ_c_, equivalent to 1/(6D_iso_) in nanoseconds, represents the overall rotational correlation time, which is calculated directly from the relaxation data. We analyzed the relaxation data with three overall models, namely, isotropic, axially symmetric, and fully anisotropic models, and subsequently, the axially symmetric model was found to best describe WW4 under different conditions.

### 4.6. NMR Characterization of ^15^N Backbone Dynamics on the µs-ms Time Scale

^15^N transverse relaxation dispersion experiments for WW4 with a concentration of 250 µM were acquired on a DRX 500 and Bruker Avance 800 MHz spectrometer equipped with a z-axis gradient cryoprobe at 25 °C. A constant time delay (*T*_CP_ = 50 ms) was used with a series of CPMG frequencies, ranging from 40, 80, 120, 160, 200, 240, 280, 320, 400, 480, 560, 640, 720, 800, and 960 Hz, with a repeat at 120 Hz. A reference spectrum without the CPMG block was acquired to calculate the effective transverse relaxation rate using the following equation:

(7) $$R_{2}^{eff}=-\ln\left(I\left(\nu_{CPMG}\right)/I_{0}\right)/T_{CP}$$

where I(ν_CPMG_) is the peak intensity in the different CPMG frequencies, and I_0_ is the peak intensity in the reference spectra.

## Data Availability

The data presented in this study are available in the manuscript.
